# Notes and News

**Published:** 1902-05-10

**Authors:** 


### Notes and News.

The annual dinner of King's College, London, will be held at The Monico on Monday, June 16th, with the Right Hon. and Right Rev. the Lord Bishop of London in the chair.

At the Westminster Hospital post-graduate clinical demonstration course, Dr. Allchin will give a demonstration on Tuesday, 13th inst., at 4.30 p.m., his subject being "Some Clinical Aspects of Arthritis."

An Exhibition of Black and White Drawings, the originals of which have appeared in *Punch*, are being shown at the Woodbury Gallery, 37 New Bond Street, for the benefit of the Hospital for Sick Children, Great Ormond Street. The exhibition will be open throughout May.

The Court of Directors of the Bank of England have granted a donation of £100 to the funds of the Middlesex Hospital in response to the appeal which the Governors have made to meet the sum of £70,000 which this old-established charity now requires to raise.

The friends and sympathisers of Dr. W. Law will hold a meeting on Friday at 2 p.m. in the King's Hall, Holborn Restaurant, for the purpose of wholly, or in some measure, relieving Dr. Law of the heavy expenses he has incurred in connection with the action recently brought against him for prescribing morphia for the relief of spasmodic asthma. Dr. Law suffers a loss of £700 although the verdict was in his favour.

A law has recently been passed in the United States levying a tax of 5d. a pound on all coloured margarine, and heavy license fees on all sellers of the same. This ought to prove an admirable way of raising revenue and doing good at the same time. The colouring of margarine is at the bottom of much fraud, and, although it is allowed by law, is a practice which should be discouraged in every way. If people are so fond of playing at eating butter as to buy coloured margarine for the purpose, they may fairly be made to pay for their strange taste, indulgence in which is the cause of much injury to the public.

Within a very short period after the recent stormy meeting at the Royal Orthopædic Hospital, the placard in front of the buildings in Oxford Street was marked with the word "Sold."

Amongst the 18 students who have entered this session at the London School of Tropical Medicine are several members of the Colonial Service, a graduate from the University of Berne, and two ladies.

A new department has been created at the Jenner Institute of Preventive Medicine, at Chelsea, for the study of the animal parasites in man and the lower animals. Major Ronald Ross, F.R.S., has been appointed head of the department.

A bill for the provision of the benefits received by English Poor Law officers under the Superannuation Act for the Scotch Poor Law officers is before Parliament. The power to retire an officer at 65 is given to the local authorities under the Bill.

The Governors of the Middlesex Hospital have received a further donation of £500 from Sir Elliott Lees, Bart., M.P., half of which sum has been allocated towards the maintenance of the inmates of the wards in the special department devoted to incurable cancer patients, and the other half to the Research Department established in connection with it for investigation into the cause of cancer.

The first course of lectures on Advanced Physiology at the new laboratories of the University of London at South Kensington will commence on May 13th, when Dr. Waller will lecture on "Signs of Life." Dr. Leonard Hill commences a course on May 14th, and various series of lectures will be given each week during the summer session. The buildings and equipments are nearly completed, and some practical work has already commenced.

It is stated in a German medical journal that although the vapour given off by burning magnesium, as used in producing "flash-lights" for photographic purposes, is not dangerous, consisting as it does of a mere oxide of the metal, the same cannot be said when the magnesium is combined with chlorate of potash. When this is done, it is stated that the ignition of the compound may give rise to the liberation of chlorine gas or some irritating chlorine compounds, by which severe illness may be caused.

Mrs. Lewis Waller's contribution of £10,000 annually to King Edward's Hospital Fund will be a very great help toward the aims of those who are trying to secure £100,000 income for the London hospitals through the Fund in the Coronation Year. Through the will of the late Mr. Lewis Waller the Fund was made residuary legatee of £250,000, but through the generosity of Mrs. Waller the hospitals will get the benefit of the money at once. It is to be hoped that the interest and energy already exhibited by the Prince of Wales since he has become president of the King's Fund, will inspire other generous persons to emulate the action of Mrs. Waller by their gifts, and to fulfil the hopes that are now entertained for the hospitals.

